# The Application of SWAT Model and Remotely Sensed Products to Characterize the Dynamic of Streamflow and Snow in a Mountainous Watershed in the High Atlas

**DOI:** 10.3390/s23031246

**Published:** 2023-01-21

**Authors:** Soufiane Taia, Lamia Erraioui, Youssef Arjdal, Jamal Chao, Bouabid El Mansouri, Andrea Scozzari

**Affiliations:** 1 Natural Resources and Sustainable Development Laboratory, Ibn Tofail University, Campus Maamora, Kenitra 14000, Morocco; 2 Institute of Information Science and Technologies (CNR-ISTI), National Research Council of Italy, 56124 Pisa, Italy

**Keywords:** hydrological modelling, satellite hydrologic products, water cycle, MODIS, SWAT, streamflow, snow, High Atlas, Oum Er-Rbia, Morocco

## Abstract

Snowfall, snowpack, and snowmelt are among the processes with the greatest influence on the water cycle in mountainous watersheds. Hydrological models may be significantly biased if snow estimations are inaccurate. However, the unavailability of in situ snow data with enough spatiotemporal resolution limits the application of spatially distributed models in snow-fed watersheds. This obliges numerous modellers to reduce their attention to the snowpack and its effect on water distribution, particularly when a portion of the watershed is predominately covered by snow. This research demonstrates the added value of remotely sensed snow cover products from the Moderate Resolution Imaging Spectroradiometer (MODIS) in evaluating the performance of hydrological models to estimate seasonal snow dynamics and discharge. The Soil and Water Assessment Tool (SWAT) model was used in this work to simulate discharge and snow processes in the Oued El Abid snow-dominated watershed. The model was calibrated and validated on a daily basis, for a long period (1981–2015), using four discharge-gauging stations. A spatially varied approach (snow parameters are varied spatially) and a lumped approach (snow parameters are unique across the whole watershed) have been compared. Remote sensing data provided by MODIS enabled the evaluation of the snow processes simulated by the SWAT model. Results illustrate that SWAT model discharge simulations were satisfactory to good according to the statistical criteria. In addition, the model was able to reasonably estimate the snow-covered area when comparing it to the MODIS daily snow cover product. When allowing snow parameters to vary spatially, SWAT model results were more consistent with the observed streamflow and the MODIS snow-covered area (MODIS-SCA). This paper provides an example of how hydrological modelling using SWAT and snow coverage products by remote sensing may be used together to examine seasonal snow cover and snow dynamics in the High Atlas watershed.

## 1. Introduction

In mountainous areas, runoff from melting snow and glaciers contributes significantly to the feeding of numerous streams, springs, and lakes [1,2]. When it gets warmer, snow that has been temporarily kept in high elevations, considered a water tower for the surrounding arid plains [2], is ultimately melted and finally finds its way to the rivers, where it plays an important role in the hydrological cycle. The majority of the rivers in the Moroccan High Atlas, including the Oum Er-Rbia, Tensift, and Sebou, have headwater catchments in snow-covered regions. According to several researchers [3,4,5], the High Atlas Mountains provide a significant percentage of the water needed for irrigation, dam storage, hydropower generation, and aquifer recharging. The hydrological analysis of such mountainous areas is more difficult and complex, due to the intricate mechanism between the snowfall and snowmelt [6,7,8]. Therefore, snowfall, snowpack, and snowmelt are among the processes with the highest impact on the water cycle in High Atlas watersheds. The inability to estimate them can lead to significant bias in hydrological simulations.

Several physically based distributed and continuous models are available for simulating the water cycle in a mountainous watershed. These models attempt to identify the driving factors present in the system and provide complete forecasts in time and space. Some hydrological models incorporate snow processes by using snow modules from a degree-day formulation to a more complicated energy balance [9,10]. These models mimic snowpack conditions to get a fair depiction of the Snow Water Equivalent (SWE) as a component of the hydrological cycle [11,12]. However, conceptual models can produce important insights at the watershed scale. These models are frequently based on a simple estimation of melt as a function of temperature and are driven by remotely sensed information or ground station data [7]. The Soil and Water Assessment Tool (SWAT) model is one of these models that has seen widespread application for simulating and forecasting the hydrological cycle, including snow processes. The SWAT model’s capacity to estimate streamflow produced by rainfall and the melting snow at the same time makes it a viable tool for use in snow-influenced watersheds [13]. It includes a snow module that generates snowpack and snowmelt based on air temperature using the index temperature method. The SWAT model is a continuous, physically based, semi-distributed, and catchment-scale model that enables the simulation of a wide number of phenomena, implemented by specific routines [14]. It has been widely used to simulate runoff, sediment transport, and agricultural chemical yields around the globe [15] as well as in the Moroccan context. For instance, Ref. [16,17,18,19,20] employed the SWAT model to simulate basin hydrology in a variety of tropical basins in Morocco and was able to demonstrate its flexibility, consistency, and applicability as a hydrological model for the planning and management of water resources. However, the majority of SWAT implementations in Morocco concentrated on low-altitude basins unaffected by snow. Only a small number of studies have expanded their scope to include downstream basins [21,22]. Nevertheless, only streamflow has been examined in these investigations. Even if the watershed is not totally dominated by snow, the impact of snow on the hydrological cycle must be taken into account for improved water management. This study looks at further determining the role played by the snow accumulation and snowmelt over a Moroccan mountainous catchment using the SWAT model.

Nevertheless, the use of such a spatially distributed model has limitations, due to the lack of in situ snow data with suitable spatiotemporal resolution. Because of the difficult landscape and the high snow thickness in mountainous regions, direct measurement of SWE is unreliable in most cases [23]. Thus, as SWE records for most of the mountainous watersheds are scarce, one of the most significant unresolved issues in hilly environments is the estimation of accurate basin-wide SWE [8]. An effective approach may consist in estimating the total snowpack by using a snow model fed by the observed remotely sensed snow-covered area (SCA) [7]. Over the last several decades, researchers have estimated SCA using data collected by optical and microwave sensors on different remote sensing satellites [24]. For regional snow investigations, the Moderate Resolution Imaging Spectroradiometer (MODIS) satellite sensors were shown to be adequate for assessing the dynamics of snowfall in the dry conditions of the Moroccan High Atlas [4]. Numerous evaluations of the MODIS product compared to other satellite-based snow products and measured snow depth have proven their excellent precision and reliability [25,26,27]. The product MOD10A1, derived from MODIS imageries, was chosen for this study because of its spatial resolution (500 m) and short revisit time (day) with an overall accuracy of 89% [28]. MODIS snow observations are increasingly being employed in hydrology, and they are especially well-suited for dispersed hydrological or land surface modelling [29]. Several studies have used SCA products derived from MODIS imageries for advancing snow and hydrological modelling [7,13,30,31,32]. For instance, Ref. [7] compared snow simulations of the SNOW17 model with MODIS-SCA and observed snow thickness in one station. Ref. [33] used MODIS-SCA to validate the snow simulation of the TRAIN hydrological model. Ref. [31] used MODIS-SCA to minimize predictive uncertainty of runoff in the Integrated Catchment-Scale Hydrological Model’s predicted streamflow (ICHYMOD). These studies support the potential of MODIS-SCA as additional data to validate or improve hydrological models, resulting in more accurate and precise discharge predictions. Only a few studies [13,30,34] have attempted to employ MODIS-SCA as a source of data on snow to evaluate or calibrate the snow simulation of the SWAT model. This paper aims at developing the use of remotely sensed data to validate hydrological models. Hence, MODIS-SCA is exploited in order to validate the SWAT model representation of the snow extent both spatially and temporally for a long continuous period.

To account for the spatial variability of the catchment, the watershed is divided into subbasins by SWAT, and these are further split into Hydrologic Response Units (HRUs) [35]. The HRUs are characterized by unique soil, land use, and slope combinations. In mountainous basins, where precipitation and temperature have a vital orographic behavior [36,37], SWAT divides HRUs into several bands, depending on the elevations, to extrapolate the meteorological forcing point [38]. Elevation bands in SWAT help to allocate the topographical influences of temperature and precipitation on the simulation of snowmelt/snowpack [11,39]. Two lapse rates, PLAPS and TLAPS, are commonly used to adjust precipitation and temperature in the SWAT model according to elevation [40]. SWAT then incorporates a snow module that uses the temperature index approach (also called the degree-day model) to generate the SWE for each elevation band, defined as the water contents of the snowpack in units of precipitation [41]. The snow temperature index method employed in the SWAT model is associated with numerous parameters, such as snowpack/snowmelt factors (SMFMX, SMFMN), snowfall/snowmelt temperature thresholds (SFTMP, SMTMP), and temperature lag factor (TIMP). However, previous studies using SWAT in mountainous watersheds included snowmelt/accumulation parameters [13,22,42,43,44]. The majority of these research projects allocated a distinct set of snow parameters to an entire basin, even though snow distribution may be very variable in space and time [45,46]. A small number of studies have attempted to assess model performance by taking into account the spatial variability of snow characteristics at various subbasins or elevation bands by using the SWAT model [47]. This research allows SWAT snow parameters to change between subbasins. The efficacy of snow parameter discretization was assessed by comparing spatiotemporal SWAT simulations using the standard lumped approach (S1) and the spatially varied approach (S2). The innovative aspect of this research is the use of remotely sensed snow cover (MODIS-SCA) outputs as an additional restriction to distinguish the differences between the two setups.

In this study, our focus is the watershed of Oued El Abid, which is a typical data-scarce catchment located in the central High Atlas and stands on an area of about 7000 km^2^. The main rivers of Oued El Abid mostly originate in mountainous areas to the piedmont plain areas, feeding Bine El Ouidane dam, one of the most important reservoirs in Morocco. Snowmelt makes a significant contribution to the river discharge, up to 50% in some local High Atlas catchments [48]. In addition to the region’s significant spatiotemporal variation of temperature and precipitation, the sparseness of the network of meteorological gauges provides strong management challenges for hydrological modelling in this region [49].

From the aforementioned discussion, the contribution and main objectives of the current paper are: (i) Developing a comprehensive SWAT-based hydrologic model for the simulation of daily snowpack/snowmelt and runoff in a Moroccan mountainous watershed; (ii) Validating the spatiotemporal snow simulation of the SWAT model by using remotely sensed snow cover products; (iii) Combining the SWAT model and MODIS imagery to analyze the snow dynamics in the region of Oued El Abid.

## 2. Materials and Methods

### 2.1. Study Area

**Figure 1:**
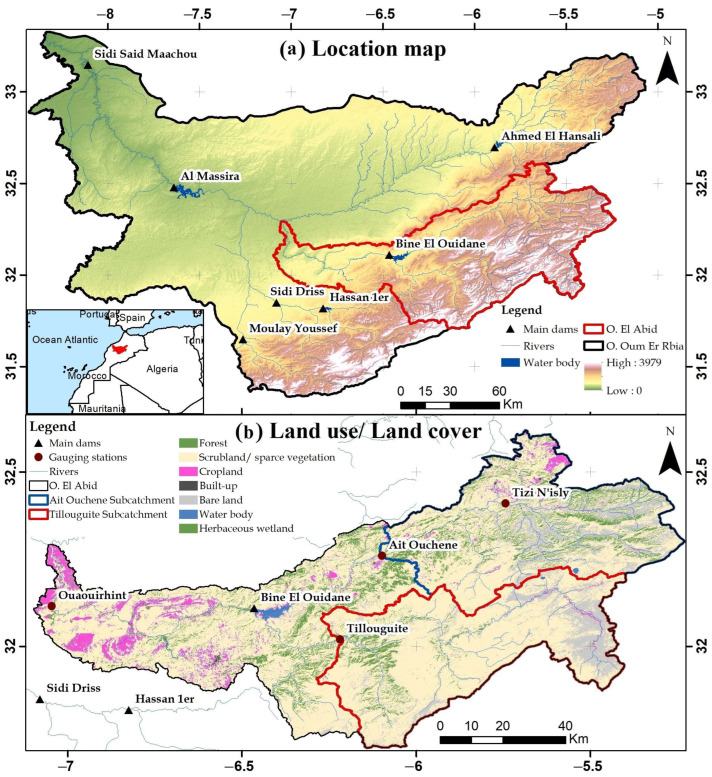
(**a**) Geographic situation of the Oued El Abid catchment. (**b**) Land use/land cover map.

The catchment of Oued El Abid is characterized by snowfall precipitation, since the highest points are located at altitudes exceeding 3000 m (Figure 1). Therefore, a great part of its area is often covered with a snowpack that persists until the end of the spring season [50]. Snowfall contributes to between 20 and 80 percent of total precipitation reported on the southern and northern parts of the High Atlas in winter, according to [51]. Later in springtime, snowmelt fills the reservoir of Bine El Ouidane after the rainy season, allowing for continuous irrigation and water consumption until the end of the growing season in May. This means that this dam plays a key role in the freshwater supply for irrigation, hydropower production, and drinking water for the people living in the central High Atlas.

The climate of the catchment is influenced by the high diversity. According to [6], streamflow variation is strongly linked to rainfall, temperature, and snow cover change. The High Atlas snow cover exhibits significant fluctuation in both space and time, which is a key feature in arid conditions [8]. Additionally, precipitation often occurs in sporadic, short events throughout a season, and even in midwinter at high elevations, moderate temperatures on a few dry days can cause complete ablation of the snowpack [49].

From a geological point of view, the Atlas domain is divided into two parts: the Middle and the High Atlas, which are essentially made up of limestone zones and cover the summits of Jbel Ghnayne and Jbel Bouykhfawne. The valley of Oued El Abid is established on a lithological diversity that develops successively in vast basins corresponding to the marly lands of the Cretaceous and crosses deep gorges, dug in the limestone series of the Jurassic and the Lias [50]. The limestones and dolomites of the lower and middle Lias, as well as the limestones of the middle Jurassic and Turonian, all exhibit a well-developed karst system in the Oued El Abid watershed. The karstic reservoirs provide a remarkable contribution to the sustainability and support of baseflow in the Oued El Abid river [52]. They also feed an array of springs that flow along the overlapping fault of the region.

### 2.2. Soil and Water Assessment Tool

In this study, the SWAT model was used to simulate hydrologic processes in the Oued El Abid watershed. SWAT is a process-based, semi-distributed hydrology and environmental quality model. For the purpose of simulating different eco-hydrological and anthropogenic processes in the research region, a daily timestep and hydrological response units are used. According to [35], SWAT simulation of a watershed is performed in two distinct phases. The first is the land phase, which controls the amount of water, sediment, nutrients, and pesticides in each subbasin. The hydrological process involved in this phase is computed using the water balance equation, including processes such as surface runoff, infiltration, soil water transport, evapotranspiration, groundwater flow, and pond contributions [41]. Secondly, SWAT simulates the routing phase to compute the movement of water, sediment, and pesticides through the channel network of the catchment. To determine the rate and velocity of flow, SWAT employs the Manning equation. The variable storage routing technique or the Muskingum river routing method is used to move water through the network of channels. Both the variable storage and the Muskingum routing methods are variants of the kinematic wave model [53]. SWAT then incorporates a snow module that uses the temperature index approach (also called the degree-day model) to generate SWE for each elevation band, defined as the water contents of the snowpack in units of precipitation. A more detailed description of the snow temperature index method adopted by the SWAT model can be found in [41], being marginal to the scope of this paper.

Most snowmelt runoff models manage spatial and temporal changes related to elevation by integrating elevation bands, which allows the model to discretize the snowmelt process depending on the topography of the watershed. Oued El Abid watershed, as presented previously, is characterized by high elevation gradients that have an extreme effect on the variability of precipitation and temperature. However, SWAT separates the watershed into subbasins, which are then further discretized into HRUs to account for the spatial variability of the catchment. The HRUs are characterized by unique soil, land use, and slope combinations. For each HRU, surface runoff and evapotranspiration estimates are determined independently, in order to provide a more accurate physical picture of hydrological processes [54]. The use of elevation bands, on the other hand, improves the modelling of the spatial variability of precipitation and temperature in mountainous catchments [41]. Based on the topographic characteristics of a watershed, the elevation bands can be used to analyze the snowmelt process. The elevation for the Oued El Abid watershed ranges between 280 m and 3687 m. Therefore, five elevation bands were incorporated in each subbasin to characterize the snowpack and snowmelt. According to [13], this number is typically sufficient. As a result, for each specific elevation band, the spatial distribution of precipitation and temperature is considered, taking into consideration two lapse rates: one for precipitation (PLAPS in mm H_2_O/km) and one for temperature (TLAPS in °C/km).

The hydrological process in Oued El Abid watershed is very complex, due to the existence of very large karst systems as mentioned in the previous section. In fact, this geological configuration is characterized by bedrock fractures, springs, and cavities. It must be underlined here that the surface runoff simulation in karst watersheds presents an important challenge because of the presence of sinkholes and other features that are randomly distributed with different characteristics (depth, diameter, infiltration rate, etc.). Several studies focused on the direct modification of the SWAT code to improve the accuracy of hydrological simulation in karst-affected watersheds [15,55]. In this work, due to the lack of observational data describing the characteristics of springs and sinkholes, we attempt to use the approach presented by [56], which introduced a technique to enhance the modelling of karstic HRUs. This technique involves activating the Crack Flow module in the SWAT model and then adjusting the SOL_CRK parameter in the calibration phase. This approach is based on the assumption that soil sinks and fissures behave like karst environments, i.e., surface features can be treated like underground features, under the point of view of the hydraulic simulation. For each soil layer, the crack volume is simulated in the standard SWAT Crack Flow module. The calculation of this volume is based on the SOL_CRK coefficient, which is estimated during the calibration phase. The SWAT model uses precipitation and temperature to estimate the flow crack volume variability [41,57] and accounts for cracks that penetrate to a depth of 50 cm in the dry season. During wet conditions or when the moisture content of the profile is above 90% of the field capacity, the amount of water flowing into the cracks is set equal to the volume of the cracks, and the rest of the water flows over the surface [41]. However, the application of the Crack Flow module was implemented in the SWAT model only on soils classified as Vertisols. Instead, in this study, the Crack Flow module was activated in all HRUs located in karst areas. A complete description of theoretical concepts for SWAT version 2012 can be found in [41].

### 2.3. Data Preparation

Considerable efforts were dedicated to data collection and pre-processing before building the SWAT input files. The model requires information about hydroclimate data (precipitation, atmospheric temperature, and river discharges), relief, soil, land use, and other complementary information.

#### 2.3.1. Land Coverage from Multi-Spectral Satellite Products

Land use/land cover (LULC) maps were produced using a supervised classification method based on a maximum likelihood algorithm. For this purpose, we used Sentinel-2 Level 2A data, which acquires 13 spectral bands with a spatial resolution between 10 m and 60 m [58]. The Sentinel-2 images were downloaded from the Copernicus Open Access Hub (scihub.copernicus.eu/dhus) provided by the European Space Agency (ESA), and they were preliminarily selected based on their cloud coverage. The Semi-Automatic Classification Plugin (SCP), a free open-source plugin for QGIS, was used in this study [59]. This plugin includes numerous tools for free image downloads, pre-processing, post-processing, and the raster calculation. The SCP allows for the semi-automatic classification of remote sensing images using several algorithms such as minimum distance, maximum likelihood, and spectral angle mapping classification [59]. Ten bands out of thirteen were selected for the classification procedure: blue (band 2), green (band 3), red (band 4), vegetation red edge (5 to 7), NIR (8 and 8A), and SWIR (bands 11 and 12), while excluding bands 1 (visible—coastal aerosols), 9 (SWIR—water vapor), and 10 (SWIR—cirrus).

The Sentinel-2 Level 2A reflectance data were used for this purpose, being already atmospherically corrected [60], and they have been resampled to a homogeneous spatial resolution of 10 m. Then, a normalized difference water index (NDWI) and a normalized difference vegetation index (NDVI) were produced and added to the ten native bands of the input dataset mentioned above. All these 12 bands were used as input to the SCP. Ground truth data for the supervised classification were randomly selected and extracted manually, based on field knowledge, pictures from field trips, and high-resolution Google Earth images. To validate the classification of LULC, 30 sites were used as ground truth reference, in order to evaluate the classification accuracy. Thus, the performance was generally very good, with a Kappa coefficient of 95% and an overall accuracy of 97%. The LULC is classified into five classes (forest, water body, matorral, agriculture, and bare soil).

#### 2.3.2. Digital Elevation Model

For the elevation data, the Digital Elevation Model (DEM) of the Shuttle Radar Topography Mission (SRTM) with 30 m resolution was obtained from the Earth Explorer portal (https:/earthexplorer.usgs.gov, accessed on 15 November 2022) of the United States Geological Survey (USGS). Several images were mosaicked and reprojected into WGS84/UTM Zone 30 system and then clipped to the study region to create a DEM raster. Each pixel in this DEM raster provides an accurate altitude estimation between 280 and 3687 m. This DEM was then utilized to define the watershed, calculate morphological characteristics of the watershed (slope and longest path flow), and generate the river network, which are used as input for the SWAT model.

#### 2.3.3. Soil Data

In this study, information about the soil was obtained from different sources. Soil texture (clay (%), silt (%), and sand (%)), organic carbon (dg/kg), and coarse fragments (cm^3^/dm^3^) were obtained from the International Soil Reference and Information Center (ISRIC), which provides a possible solution for an accurate representative global soil database at a recommended fine resolution [61] for modelling purposes. It represents a set of updatable soil characteristics and class maps of the world at a spatial resolution from 250 m to 1 km [62]. Regarding the electrical conductivity of the soil, data were derived from the field work of [63], by interpolation of the samples using normal kriging and then feeding the data directly to the HRU files. The soil erodibility factor K_USLE was estimated using the equations proposed by [64], which require information about soil texture and organic carbon content. The available water content and the hydraulic conductivity are estimated using a field and pond hydrology program called Soil–Plant–Atmosphere–Water (SPAW) [65].

#### 2.3.4. Hydroclimatic Data

Daily precipitation datasets, covering the period 1980–2015, were obtained from three meteorological stations located within the catchment (Tillouguite, Tizi N’isly, and Ouaouirhinnt). The values of the minimum and maximum temperature on a daily basis were obtained from the instantaneous measurements provided by the climatological forecasting system called CFSR (Climate Forecast System Reanalysis). CFSR data cover the period from 1979 to 2014 with an almost global coverage, with gridded data having a spatial resolution of 0.5 degrees [66]. Observed daily streamflow timeseries, required for model calibration and validation, were obtained from the Hydraulic Basin Agency of Oum Er-Rbia. These data concern three streamflow stations, Tillouguite, Ait Ouchene, and Tizi N’isly. The records of daily inflow to Bine El Ouidane dam are estimated by using water balance from the measured monthly volume, evaporation, and water extraction from the reservoir. Then, they were used to calibrate and validate model inflow simulations. The parametrization of input data was processed using the ArcGIS-ArcSWAT interface for SWAT 2012. The characteristics of the reservoir impoundment, such as the surface area, the volume of water needed to fill the reservoir to the emergency spillway, and initial reservoir volume, were given as input directly to the SWAT files describing the reservoir.

### 2.4. MODIS Snow Coverage Data

#### 2.4.1. Data Availability

Given the unavailability of in situ snow data in our study area, we used the remotely sensed snow cover products derived from MODIS. Several products were developed and launched using Aqua and Terra MODIS data. The product MOD10A1 was chosen for this study because of its high spatiotemporal resolution at 500 m and daily timestep beginning from 24 February 2000 [67]. This product was generated using the SNOMAP algorithm developed by [68]. The SNOMAP algorithm for snow mapping primarily relies on the Normalized Difference Snow Index (NDSI) [67]. The NDSI calculates the magnitude of the difference in reflectance between one visible band (green) and a shortwave infrared (SWIR) band. The NDSI score varies from −1 to 1, representing the theoretical range for snow. A pixel value with an NDSI above 0 is considered to hold some snow, whereas a pixel with an NDSI below 0 is snow-free land [67]. SNOMAP considers a snow pixel as having an NDSI higher or equal to 0.4.

The MODIS-Terra-based product MOD10A1 is accessible by the National Snow and Ice Data Center (NSIDC) web archive “https://nsidc.org/data/mod10a1/versions/6 (accessed on 5 June 2022)”. In addition, the product was archived by Google and connected to the cloud-based geospatial platform Google Earth Engine (GEE) for open-source use (GEE snippet: ee.ImageCollection (“MODIS/006/MOD10A1”)). The MOD10A1 product includes NDSI snow cover, snow albedo, fractional snow cover, and quality assessment (QA) data. Possible values of the NDSI snow cover product of MOD10A1 are summarized in Table 1.

#### 2.4.2. Cloud Removal Procedure

Cloud obscuration remains the main obstacle to using MODIS snow cover products. The frequent presence of clouds in MOD10A1 covers substantial portions of the collected scenes, up to 30% in the High Atlas during the wet season [7]. Consequently, many data are missing. Various algorithms and methods have been introduced to remove clouds in MODIS products [5,69,70]. In this study, a spatiotemporal filter from [71] is used to enhance the number of useable images and improve snow/cloud classification. The code is written in Python, and it is freely available at “https://gitlab.inf.unibz.it/earth_observation_public/modis_snow_cloud_removal (accessed on 13 June 2022)”.

However, MOD10A1 data from 24 March 2000 to 31 December 2015 were obtained from the NSIDC web archive. This corresponds to 5790 dates, of which 5732 (99%) are available for MOD10A1 for the study area. We reclassified the different classes in the original product MOD16A1 to three main classes: no-snow (Inland water, Ocean), snow (NDSI snow cover), and no-data (missing data, clouds, fill, saturated detector, and no-decision). The reclassified product was corrected using the cloud removal code. The used algorithm offers a cloud removal procedure, consisting of a sequence of spatiotemporal filters [71], which basically follows that applied in [59,72,73,74]. These steps are as follows:

- The initial step of this procedure is to pre-process the data to reduce the edge effects of snow and cloud detection in MOD16A1. A simple mean filter was employed to reclassify only snow or cloud pixels depending on their surroundings. Within a user-defined grid, if the surrounding pixels had more snow pixels than cloud pixels, the pixel is reclassified as snow; otherwise, the pixel is reclassified as cloudy.
- The second phase involves creating a conservative temporal filter based on the assumption that no change should occur within a short period (i.e., detected changes can be due to clouds only). In this phase, a cloudy pixel is reclassified as snow if there was snow the prior or the next day at the same pixel. If the previous or following days were cloudy, values from two days before or after are also examined. If both were snow, the pixel is turned to snow; otherwise, the pixel is left as cloud.
- The third stage consists of applying a spatial filter, which takes advantage of the elevation dependency of the snow cover. On any given day, the mean elevation of all snowy pixels is calculated. Then, all cloudy pixels with an altitude above this elevation are reclassified as snow.
- Finally, a fourth step consists of a greedy temporal filter replacing the rest of the cloudy pixels with the nearest non-cloudy ones. In this step, the next non-cloudy pixel, either forward or backward in time, was employed to fill the cloudy pixel. If the next non-cloudy pixel was an equal number of days in both directions, the backward value was used.

### 2.5. Model Performance, Calibration, and Validation

#### 2.5.1. Performance and Uncertainty Analysis

The calibration and validation processes have been performed with the aid of the complementary software package SWAT-Calibration and Uncertainty Programs (SWAT-CUP) [75]. Sensitivity analysis was done simultaneously during calibration [75]. The Sequential Uncertainty FItting algorithm version 2 (SUFI-2) was used to calibrate the model [76], while Nash–Sutcliffe Efficiency (NSE) and Percent Bias (PBIAS) metrics were used to assess the model performance [77].

To account for those uncertainties, it is essential to implement an uncertainty analysis to increase confidence in numerical modelling. The 95% prediction uncertainty (95PPU), which is derived at the 2.5% and 97.5% levels of the cumulative distribution of output variables generated from Latin hypercube sampling, is used in SUFI-2 to quantify the model output uncertainty [75]. The quantification of the fit between the simulated and observed data, expressed in 95PPU (95 Percent Prediction Uncertainty), can be achieved by using two statistical criteria: (i) the p-factor expresses the percentage of observations within the 95% confidence interval; it is a normalized factor varying from 0 to 1, where 1 corresponds to a perfect correlation between the data; (ii) the r-factor expresses the average width of the confidence interval band 95PPU, divided by the standard deviation of the observations. It varies from 0 to ∞, where a value close to 0 indicates very good performance of the model.

#### 2.5.2. Model Calibration and Validation

**Figure 2:**
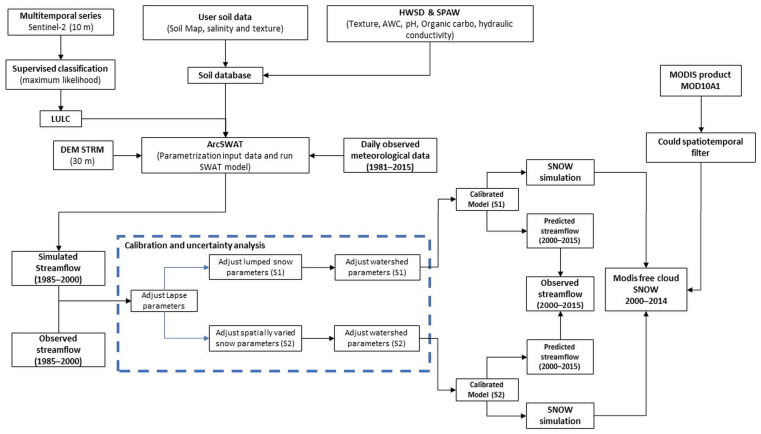
Flowchart of the methodology followed in this work.

The SWAT model was calibrated and validated at four flow gauging stations for the periods 1985–2000 and 2001–2015 based on the available observation of streamflow. Three years of warm-up (1982–1984) were considered. In terms of calibrated parameters, we took into account the lapse, snow process, and watershed parameters that are most sensitive to streamflow based on a global sensitivity analysis made by using SWAT-CUP. Following the suggestions found in [78], PLAPS and TLAPS, the parameters that control the spatiotemporal distribution of precipitation and temperature, were the first parameters to be calibrated and locked at their optimal values before being removed from further calibration. In the following step, we consider snow parameters. Most users assumed a lumped value for all subbasins, which is the default configuration of the SWAT model. However, because snow parameters depend on local characteristics such as vegetation, wind speed, relative humidity, and aspects which are not unique among the such large-scale regions, this technique may fail to give a realistic simulation of snow processes. In reality, the SWAT model allows for the definition of either a singular set of snow parameters for the whole basin or a separate set of snow parameters for each subbasin. Accordingly, the lumped approach as well as the spatially varied approach have been compared to assess the effectiveness of the snow parameter discretization. In the lumped model, the snow parameters are adjusted for the whole catchment (S1); instead, the spatial approach suggests that snow parameters will vary at the subbasin level (S2). Finally, the remaining watershed-related parameters were calibrated in the last step for both S1 and S2 (Figure 2).

## 3. Results

### 3.1. Streamflow Performance

After setting-up the SWAT model and specifying the experimental design, the model was calibrated and validated using measured streamflow at four gauging stations (Tillouguite, Tizi N’isly, Ait Ouchene, and Bine El Ouidane). Each hydrological station was calibrated independently by changing the parameters linked to its upstream subbasins. Once the optimal parameters are validated, their values are fixed before moving to the next stations by varying only the parameters at the level of the intermediate subbasins. This approach increases the degree of parameter freedom compared to the single calibration [79], despite the fact that this process requires more time and a larger number of simulations. Thus, for each station, we proceeded with three to five iterations of 600 simulations for calibration and one iteration of 600 simulations for validation. Table 2 provides a summary of the respective goodness-of-fit statistical results. However, the NSE between the simulated and observed reveals that model S1 is satisfactory calibrated at all gauging stations. The calibration of Tizi N’isly (0.51) and Tillouguite (0.42) performed poorly when compared to Ait Ouchene (0.59) and Bine El Ouidane dam (0.60). Model S1 has not been able to validate streamflow, particularly in Tizi N’isly (0.33) and Tillouguite (0.44). Indeed, NSE values have improved significantly in calibration and validation at all gauging stations when considering the S2 model. This fact is observed in the overall performances. The values of NSE and PBIAS were found at 0.53 and −15.23%, respectively, indicating an acceptable performance of S1 in calibrating daily streamflow, while in the validation phase, NSE decreased to 0.46. However, S2 has improved the results in calibration and validation compared to S1. According to [77], an NSE value of 0.5 can be satisfactory when considering daily simulations. Therefore, the statistical criteria obtained in validation make for strong evidence of the robustness of the best-compromise ranges.

Any investigation with the calibrated model must include uncertainty analysis in the result [40]. Therefore, it is necessary to implement uncertainty analysis to gain more confidence. The SUFI-2 approach is used to assess the SWAT model’s uncertainty, which is then stated in terms of parameter uncertainty. To ensure that the measured data first fall inside the 95PPU, calibration starts with the assumption of a substantial parameter uncertainty before gradually reducing it. The majority of the data have to be bracketed inside the 95PPU by balancing the p-factor and r-factor while aiming for the smallest uncertainty band. In Table 2, the p-factor and r-factor measure the uncertainties in outputs when calibrating snow parameters under both scenarios. Scenario S2, after the same number of iterations, has increased the percentage of observations bracketed by 95PPU with the trade-off of larger uncertainties. However, in high-quality input datasets, the p-factor should be in the range of 0.8 to 1, but for low-quality datasets (i.e., with a considerable presence of missing data and outliers), it may be adequate to consider a p-factor over 0.5 [76]. Visual evaluation of streamflow simulations as well as parameter uncertainties can be found in Appendix A.

### 3.2. Evaluation of Snow Simulation Using MODIS-SCA

#### 3.2.1. Areal Coverage by MODIS and Its Relationship with SWE

Usually, during the calibration of the SWAT model, the simulated discharge is compared to the observed one. SWAT uses a temperature index to simulate snow accumulation and snowmelt at the level of elevation bands in terms of SWE. On the other hand, MODIS measurements do not give information on the quantity of SWE in the watershed as they only offer spatial information about the presence of snow and its distribution. Snow models would benefit from SWE more than SCA, but unfortunately, the precision of remote sensing SWE data is too poor to be used adequately [80,81]. For this reason, conceptual degree-day snow models are frequently calibrated using observed streamflow [82]. Therefore, in this study, all of the model’s parameters, including the lapse and snow parameters, were optimized by using the streamflow. To quantify the hydrological model’s capabilities in simulating the snow cover, researchers commonly define a snow depth (referred to as the SWE-SCA threshold) above which coverage will always extend 100% of the area. SWE-SCA thresholds were utilized to convert SWAT-model-simulated SWE to SCA to fully utilize MODIS-SCA data at the catchment size. However, spatially varying thresholds were applied to transform the simulated SWE by the SWAT model to SCA. A range between 5 and 35 mm was applied in each subbasin to obtain the best fit between the model and the remote sensing product MODIS-SCA.

Two assessment indices, namely NSE and PBIAS, were utilized to compare the simulated SCA and MODIS-SCA at daily, weekly, and monthly timesteps from 2000 to 2015. The thresholds were fixed to meet the best fit between observation and model in each subbasin. Table 3 represents the thresholds that best fit the observations. In S2 simulations, the threshold SCA-SWE was found to be higher compared to S1. This is because of the higher amount of SWE generated in this model compared to S1. When comparing values obtained at each area, we notice that Tillouguite has a slightly larger threshold. As a result, the threshold has shown high sensitivity to the output. Furthermore, this value should vary spatially to get the best estimates of SCA over large-scale basins.

#### 3.2.2. Temporal Evaluation of MODIS-SCA vs. SWAT-SCA

In this section, we assess the SWAT model’s snow simulation on the temporal scale. Timeseries of SCA from MODIS and SWAT between 2000 and 2015 were derived for the sub-catchments Tillouguite and Ait Ouchene, as well as for the whole watershed of Oued El Abid. The goodness of fit between the timeseries were evaluated using NSE and PBIAS. The results, demonstrated in Table 4, indicate that the NSE values vary between 0.53 and 0.73, with a PBIAS value around −14% for the Tillouguite sub-catchment, suggesting a satisfactory performance of the S1 model in simulating SCA in this sub-catchment. When considering the basin-wide SCA, the model has shown a relatively high negative bias with a satisfactory simulation of the temporal variation, demonstrated by acceptable NSE values. In contrast, the simulation of SCA by S1 in Ait Ouchene illustrated lower accuracy, with negative values of NSE along with a significantly high negative bias. However, the S2 model has been able to slightly increase NSE values and reduce the bias between simulated SWAT-SCA and MODIS-SCA. For instance, in the Ait Ouchene sub-catchment, model S2 reduced the bias from −171 to −21 and increased NSE values significantly in this sub-catchment from −0.4 to 0.14 in the daily timestep. When comparing SCA from both SWAT and MODIS, despite some misestimation of SCA, particularly in the Ait Ouchene sub-catchment, the results showed a very good agreement generally in both visual and statistical analysis. This means that the SWAT model has been able to suitably simulate the temporal fluctuation over the catchment.

**Figure 3:**
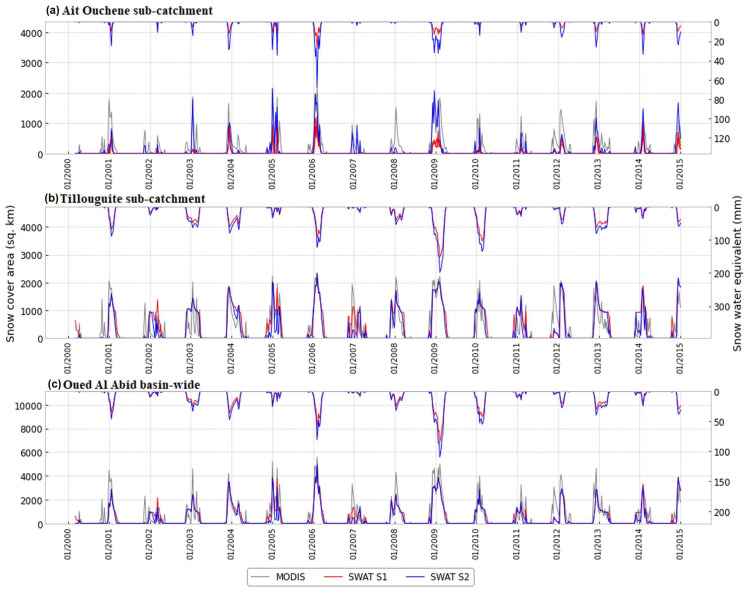
Weekly evolution of MODIS-SCA, SWAT-SCA, and SWE in the sub-catchments of (**a**) Ait Ouchene, (**b**) Tillouguite, and (**c**) Oued El Abid basin-wide.

Figure 3 shows the temporal evolution of daily SCA simulated by SWAT models against MODIS-SCA over the sub-catchments of Tillouguite and Ait Ouchene and the basin-wide sub-catchment of Oued El Abid. Along with SCA, Figure 3 also shows the evolution of SWE simulated by the SWAT model. From Figure 3, we can observe a good visual agreement between MODIS-SCA and SWAT-SCA. Moreover, for most years, SWAT and MODIS seasonal snow begins and ends simultaneously. Thus, the graphical analysis confirmed the aforementioned statistical criteria. It can be observed from the figure that the SWAT model has been able to reproduce SCA more accurately in Tillouguite than in the Ait Ouchene sub-catchment, where the simulation of several seasons was badly underestimated by SWAT. However, S2 improvement of the SCA simulations in Ait Ouchene has been observed particularly in 2004/2005, 2005/06, and 2008/09. In contrast, the basin-wide SCA is well represented in most snow seasons with respect to the temporal variation. In general, the dynamics of SCA are characterized by extremely high inter-annual variability in maximum snow cover, occurrence, and snow season duration. Both models represented well the peaks that happened in the seasons of 2004/05 and 2005/06, in addition to 2008/09. Furthermore, the amount of SWE generated by the model has been increased with S2, particularly in the Ait Ouchene sub-catchment. This might be due to the higher snowfall/snowmelt factors, which allow for higher snow accumulation and slower melting.

#### 3.2.3. Spatial Resolution

**Figure 4:**
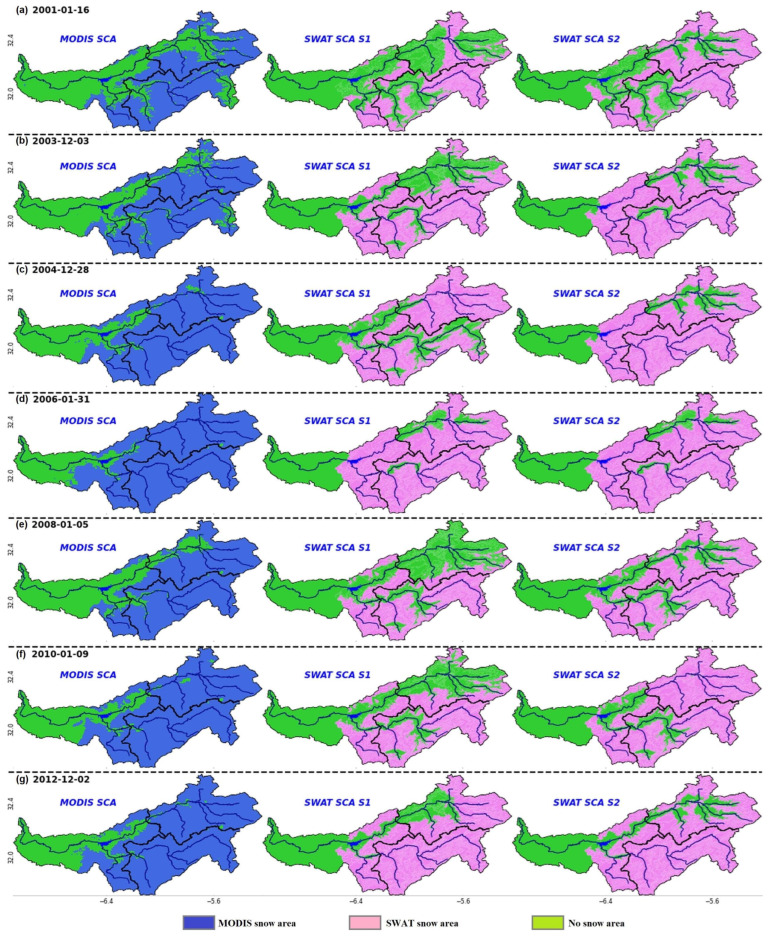
Snow extent visualizations for Oued El Abid catchment from MODIS and SWAT. (**a**) 16 January 2001, (**b**) 03 December 2003, (**c**) 28 December 2004, (**d**) 31 January 2006, (**e**) 5 January 2008, (**f**) 9 January 2010, (**g**) 2 December 2012.

This section analyzes the spatiotemporal representation of snow simulated by SWAT models and compares results with MODIS-SCA. The SWAT spatial representation of snow is generated at the elevation band scale. While MODIS-SCA offers gridded data with a 500 m resolution. Figure 4 illustrates the spatial distributions taken at similar times during winter, corresponding to the day of the maximum SCA detected by MODIS. The figure concerns only the humid seasons of 2000/01, 2002/03, 2003/04, 2005/06, 2008/09, 2010/11, and 2013/14. It can be seen that the SWAT models fairly simulated the extent on the day of maximum extent detected by MODIS in most cases. However, in some seasons, such as 2001, 2003, 2008, and 2009, the simulation by S1 did not reproduce the extent very well compared to S2, especially in the northern part of the catchment, which is underestimated.

**Figure 5:**
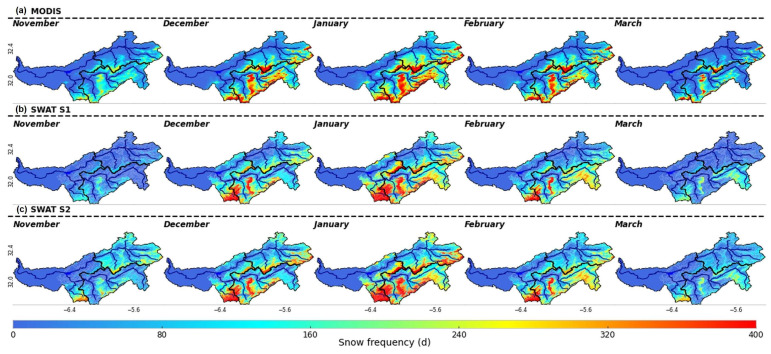
Occurrence frequency of snow during the period between 02/2000 and 12/2014. (**a**): MODIS, (**b**): SWAT S1, (**c**): SWAT S2.

Figure 5 explores the occurrence frequency of snow during the period of study (2000–2014) as simulated by the SWAT model compared to the MODIS snow occurrence. By comparing the maps in the figure, we can observe a very high similarity between SWAT models and MODIS in terms of the spatial distribution of snow occurrence. SWAT model snow occurrence in the study area has lower estimates than in MODIS but maintains a similar distribution, which obeys the intuitive rule that higher snow occurrences are at higher altitudes. The similarity between MODIS and SWAT models is also demonstrated at the temporal scale. Therefore, both representations suggested that the majority of the snow occurred in the three months of December to February, while November and March receive less snow. Despite that, SWAT models have underestimated the frequency of occurrence compared to MODIS in all areas, particularly in November and March, when we can observe the clear discrimination of snow by the S1 model, more evident in the Ait Ouchene sub-catchment. This means that snow begins late and melts early according to S1. In fact, after using spatially varied snow parameters (model S2), the model was flexible enough to pay off differently for missing processes in the northern part of the basin, resulting in a good simulation of snow cover in most of the months. Moreover, S2 shows more snow occurrence during November and March than S1. The disparity between S1 and S2 indicates that a single set of snow parameters for the entire basin may not accurately mimic the natural snow processes of each subbasin. Basin-scale snow conceptualization is appropriate for river basins with homogeneous topography and identical snow behavior throughout all subbasins [83]. By varying snow parameters, the model resulted in positive feedback, as it enabled consistent simulation of both variables: SCA and streamflow.

#### 3.2.4. Elevation vs. Snow

**Figure 6:**
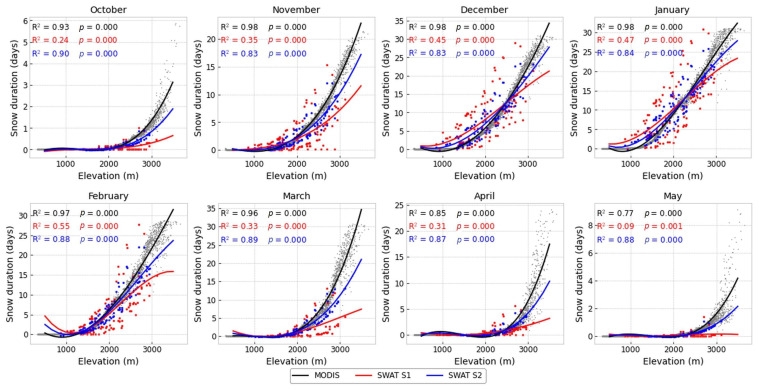
Relationships between altitude and snow duration from MODIS (black), SWAT S1 (red), SWAT S2 (blue).

The effect of elevation on snow cover during the snowfall season, as it is simulated by the SWAT models and obtained from MODIS, is shown in Figure 6. The illustration represents the curve fittings of snow duration (days) with elevation (m) during the period of 2000–2014. All the fittings in this analysis passed the F-test (*p* < 0.01). Snow durations from MODIS exhibit a strong relationship with elevation, easily identified by fitting polynomial functions of order 2. There is a clear monthly pattern of snow cover occurrence, where the topographic control of snowfall is shown. However, the relationship between snow duration and elevation is not linear but shows that snow duration begins slowly at lower elevations and rapidly speeds up when elevation is above 1500 m. As stated by [7], the majority of the snowfall in the Oum Er-Rbia basin, even at its peak, is localized above 2500 m. Ref. [6,84,85] reported similar findings, demonstrating that snow variation in the High Atlas is largely dependent on elevation. Generally, SWAT models and MODIS are in agreement about the distribution of order between months, as both datasets show more snow in January, followed by December and February with similar distribution. Despite that, results illustrate remarkable differences in the shape of the elevation–snow duration relationships between SWAT and MODIS, particularly in October, March, April, and May. Model S1 demonstrated a poor correlation in accumulation and ablation months. Model S1 predicts more snow days in low-lying areas and fewer at higher elevations from November to February. This might be caused by the lumped approach used in this model, which suggests using a unique set of parameters that control snowfall and snowmelt processes. S1 tends to underestimate average snow duration in most cases. In contrast, the S2 model retains greater relationships between elevation and average snow duration throughout all months studied. However, the S2 model corrected snow duration at low and high elevations, demonstrating good improvements.

## 4. Discussion

Hydrological modelling in a karst environment characterized by a high range of altitudes and mostly fed by snowmelt is a very challenging task, implying a strong attention to both conceptual and data processing aspects. In this work, various specific strategies for feeding the hydrological model in an effective way have been tested. In particular, the snow parameters in SWAT are permitted to be spatially distributed among the subbasins, in order to address the geographical variability of snowmelt/accumulation processes. The efficacy of snow parameter discretization has been evaluated by comparing the standard lumped approach (S1) and the spatially varied approach (S2). The spatiotemporal simulation of snow was validated using MODIS-SCA.

### 4.1. The SWAT Spatial Representation of Snow and Its Evaluation Using Satellite Snow Coverage from MODIS

The snow module in the SWAT model calculates SWE for each elevation band inside each HRU. The width of elevation bands is considered the maximum spatial resolution of the model. However, the higher the number of HRUs (which are user-defined), the greater the computational demands. At each HRU, users are allowed to define up to 10 elevation bands within each HRU. Elevation band ranges can be defined as uniform or different, with thinner bands at higher elevations [13]. Moreover, the SWAT grid [86] was developed to run landscape simulations on a regularized grid using a modified landscape routing method to overcome the spatial limits of the HRU approach. The HRU representation was chosen for this investigation because of the low processing needs of the gridded version [87]. SWAT allows for the snow parameters to be varied in the level of elevation bands, giving them full freedom. As proven by this work, parameter flexibility comes with bigger uncertainty trade-offs (Table A1). Intuitively, calibrating spatially distributed models with only one point of streamflow does not ensure that the hydrological components are accurately simulated in all parts of the study area. To address this limitation, the use of the remote sensing product MODIS-SCA has allowed the spatial evaluation of SWAT model simulations of seasonal snow dynamics at its finite entity in the mountainous Oued El Abid watershed under both scenarios (S1 and S2). However, results indicated that a lumped set of snow parameters for the whole watershed may not effectively simulate the natural snow dynamics of each subbasin, as shown by the discrepancies between S1 and S2 (Figure 5). The model produced positive feedback by modifying snow parameters, since it allowed for consistent modelling of both variables: SCA and streamflow. Despite the occasional underestimation of SCA, notably in the Ait Ouchene basin, when comparing SCA from SWAT and MODIS, the findings demonstrated very good agreement in both visual and statistical analyses (Table 4). This indicates that the temporal variability over the catchment has been adequately simulated by the SWAT model.

### 4.2. SCA-SWE Relationship

To assess the hydrological model’s ability to simulate snow cover, SWE-SCA thresholds were used to convert SWAT-model-simulated SWE to SCA. Various authors have tested the SWE-SCA threshold relationship in the literature [13,30,88,89]. However, to convert the SWAT model SWE to SCA, Ref. [13] used a fixed threshold. The use of this fixed threshold prevents more complex snow depletion curves that necessitate the inclusion of additional unknown factors [90,91]. For validating the snow spatial distribution, instead of a constant SWE-SCA threshold, Ref. [30] used varying thresholds. According to their findings, the relationship between SWE and SCA varies depending on whether a snowmelt phase or a snow accumulation period is examined. Snow accumulates irregularly in the watershed because of wind drift redistribution, and in addition, snow melts heterogeneously depending on the mountain slope and its direction [92]. Nevertheless, Ref. [93] stated that varying a SWE-SCA threshold between 20 mm and 50 mm in a distributed energy balance model does not significantly influence the outcomes. In our study, the threshold has shown high sensitivity to the output and should also vary spatially to get the best estimates of SCA over large-scale basins (Table 3).

### 4.3. The Integration of MODIS in the SWAT Model

However, MODIS snow products were basically employed to assess the depiction of snow cover extent provided by the simulation of the SWAT model snow routine, in addition to the standard calibration and validation based on discharge. Future investigations are needed, in particular concerning incorporation of this information into the SWAT model. Calibration and assimilation as well as simple integration can be used to incorporate MODIS information into hydrological models to improve the water balance simulation in SWAT. For example, Ref. [94] used a direct insertion of snow cover observation and other information to mimic snow processes in the gridded version of the SWAT model. Furthermore, Ref. [30] employed two separate units, MODIS-SCA and SWAT SWE, to manually modify snow parameters by checking for consistency between the two timeseries. This calibration process provided an excellent illustration of how to modify the model snow parameters by using snow measurements instead of runoff, which is a promising and helpful approach for ungauged watersheds. Nevertheless, this method assumes that SWE and SCA have linear relationships, which is not the case. In reality, the relationship between SWE and SCA varies depending on whether a snowmelt or accumulation phase is examined [89,90]. Furthermore, MODIS snow cover data have also been used to create snow depletion curves in hydrologic investigations and regional modelling [95,96]. These depletion curves can be used to improve SWAT simulations. However, many researchers have attempted to reconstruct SWE from satellite remote observations without the need for in situ data. For example, Ref. [97] used downscaled forcings and satellite-based estimations of fractional snow-covered area (fSCA) to build up the snowpack in reverse, from melt out to the peak. This approach has been demonstrated to reliably predict SWE in mountain ranges across the world [98,99]. These estimates could be used to calibrate SWE simulations of the SWAT model. Ref. [44], for example, studied the influence of reconstructed SWE on streamflow and SWE forecast accuracy in the SWAT model. The remote-sensing-based SWE reconstruction product is highlighted in this work as a possible alternative solution for model calibration in ungauged snow-influenced watersheds. Moreover, different snow processing methods, rather than simple degree-day models, such as the Energy Mass Balance model, could be coupled with SWAT to improve the simulation of SWE as well as of streamflow. Since the Energy Mass Balance model is equipped with more sophisticated physical processes [100,101], future research on improving the SWAT-SWE simulations should aim to incorporate an improved Energy Mass Balance model into the SWAT snow algorithm.

### 4.4. Snow Dynamics over the Studied Area

**Figure 7:**
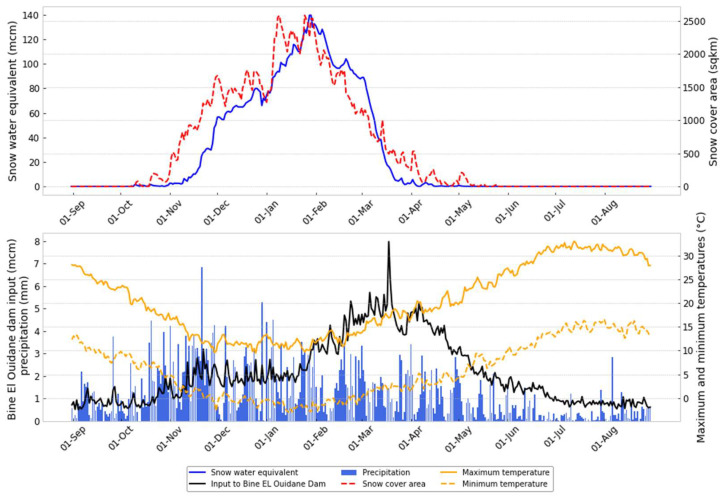
Daily average evolution of snow water equivalent, snow cover area, streamflow, precipitation, and temperature for the period from 2000 to 2014.

**Figure 8:**
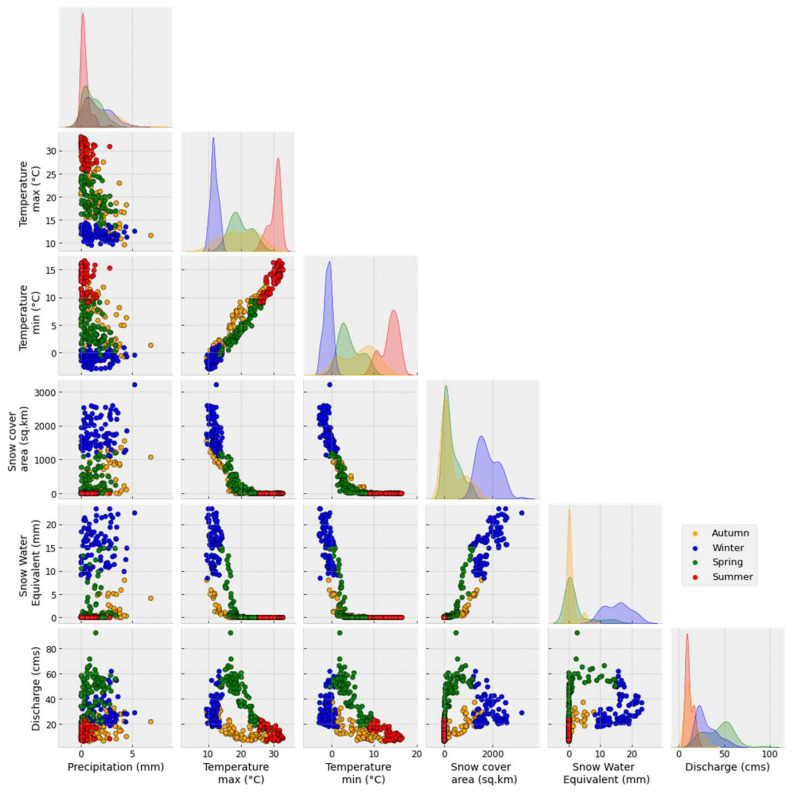
Correlation between SCA, SWE, discharge, precipitation, and maximum and minimum temperature.

**Figure A3:**
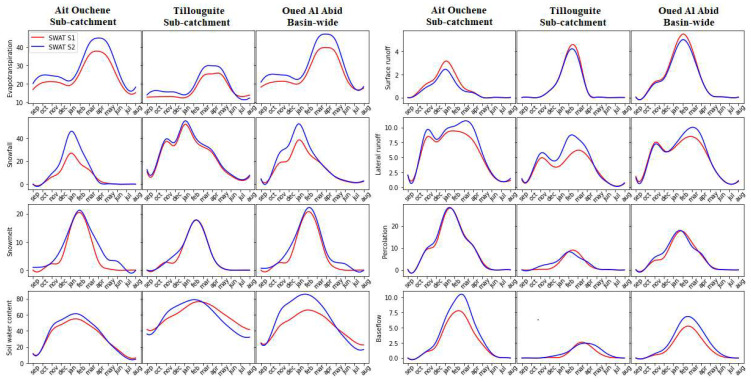
Impact of SWAT configurations on simulated water balance components (mm).

In this section, we use the SWE estimation from the SWAT S2 model, SCA from the MODIS products, and hydrometeorological observations to provide some insights into the water dynamics in this region. Therefore, Figure 7 illustrates the average daily variation of different components for the period from 2000 to 2014. In addition, Figure 8 illustrates the scatter plots between different variables. Extremely high inter-annual variability in the maxima, distribution, amplitude, and duration of the snow season characterizes the dynamics of the SCA and SWE in the region (see Figure 3). Indeed, it is found that the snow season occurs between November and April for Oued El Abid (Figure 7). The model predicts a slow build-up of the SWE, beginning in early November and reaching a peak around the beginning of February (Figure 7), followed by melting that ends in late April (see Figure A3). Similar findings were stated by [8,93]. Maximum SWE, as simulated by the SWAT model, reaches 140 million cubic meters (mcm) on the basin-wide scale (Figure 7). However, Ref. [7] estimated an SWE peak of 150 mcm in the whole catchment of the Oum Er-Rbia basin. The Oued El Abid catchment comprises a large portion of Oum Er-Rbia; it is entirely located at high elevations, where snow is common. Furthermore, the observed inter-annual fluctuations in SCA and SWE are influenced by topography (see Figure 6). Therefore, the majority of the snowpack is located above 2500 m, covering around 80% of the basin, while locations below 2000 m have few snow occurrences. Melt, on the other hand, occurs swiftly, primarily between February and May (Figure A3), when temperatures increase. The model also predicts non-negligible melt rates throughout the snowy season, even in January (see Figure A3). This occurs as a result of the abundance of warm days throughout the winter (Figure 7). The inter-annual variability detected in SWE and SCA affects the fractions of snowmelt contributing to streamflow, suggesting that melting snow will be variable from one year to another. According to [8], between 15% and 51% of streamflow is contributed by snowmelt, making the snowmelt in the High Atlas the principal supply of water for the Bine El Ouidane dam. Hence, the role of snowfall/snowmelt in feeding the dam is very crucial. Any changes within the snow processes will impact the sustainability of agricultural activities surrounding the dam. Additionally, it is evident that SCA variations, which are influenced by air temperature, have an impact on the hydrologic response (Figure 8). However, the impact of severe climate alterations, including frequent drought events, increasing temperature, and reduced precipitation, has been reported recently in this region. Evidence from historical climatic data suggested some significant increases in temperature and reduction in precipitation [102,103]. According to [104], an increase in temperature favors rain-on-snow events, which in turn provide high runoff. A high runoff coefficient can be very damaging to the public and private infrastructure and cause many victims [105]. In addition, a rise in temperature will cause a reduction in snowpack and snowmelt mainly at low altitudes. Ref. [104] suggested that by the end of the century, snow may become a rarity below 2000 m. This will seriously harm the region’s economy, which is concentrated on agricultural activities, and endanger the way of life for millions of small-scale farmers.

## 5. Conclusions and Limitations

This paper illustrates a synergistic use of the remote sensing product MODIS-SCA and the SWAT hydrological model for the assessment of seasonal snow dynamics and runoff in a mountainous catchment. In this paper, attention is focused on the Moroccan High Atlas catchment. Snow study in this region is a difficult task, due to the very variable topography and the resulting significant fluctuation in snow cover, which is even worsened by a lack of snow observations in the area. Remotely sensed MODIS snow cover products were chosen as areal snow coverage observations to illustrate geographical and temporal patterns of snow distribution, and they were also intended to assess snow simulations of the SWAT model. The calibration operation was processed using the SUFI-2 algorithm within the SWAT-CUP program. After the initial setup of the sensitive model parameters, an automatic calibration was performed using the daily streamflow.

To address the spatial heterogeneity of snowmelt/accumulation parameters across subbasins, SWAT snow parameters are allowed to be regionally scattered throughout the subbasins in this study. The spatially varied method (S2) and the lumped technique (S1) were compared in order to assess the effectiveness of snow parameter discretization. Although certain peaks were accurately captured by both SWAT models, others were not. As a general result, the S2 model exhibited a clear enhancement in both the recession limb and in the base flow estimation. As a result, S2 was more consistent with observational streamflow and MODIS-SCA.

In general, SWAT proves that it can accurately estimate streamflow. Additional research is still necessary to address the parameter uncertainties. Additionally, the fitted models reproduce most of the properties of the snow cover distribution determined from the MODIS data at the basin size. The amount and fluctuation of snow cover are well-approximated on a daily, weekly, and monthly timescale, confirming the SWAT spatial representation of snow presence with a general good quality. When employing spatially variable snow parameters (S2), the SWAT model was flexible enough to compensate for different missing processes in the northern portion of the basin, leading to a satisfactory simulation of snow cover in most of the months. As a consequence, the model generated good feedback, since it made it possible to consistently simulate both the SCA and the streamflow variables.

The findings of the synergy between remote sensing and modelling, on the other hand, are encouraging; nonetheless, in situ measurements for validation and model bias correction are still required. It is also important to realize that MODIS-SCA is a poor indicator of snowpack extent. No missing data does not mean error-free data, and the filtering process may fail in some instances [84]. Several studies have shown high accuracy [5,13,28], but they also identified some major limitations. For instance, MODIS products do not provide any information on the amount of SWE present in the watershed; they only provide spatial information regarding the presence or absence of snow. Moreover, the monitoring of snow may be very sporadic in areas with high cloudiness. The close spectral signature in the visible domain could lead to some misclassification between snow and cloud. Furthermore, Ref. [106] revealed that MODIS-SCA is not efficient in capturing the local fluctuation of SCA, related to the terrain aspect effect caused by solar radiation. However, as long as ground station data are scarce, any estimate of snow dynamics in the High Atlas will be highly unreliable. Therefore, it is critical to establish a snow measuring network, particularly for direct SWE snow depths over the Moroccan Atlas watersheds. More direct observations of latent heat fluxes, in particular, are required to validate sublimation and modelled fluxes in this region [7].

## Figures and Tables

**Table 1:** NDSI snow cover dataset descriptions.

Value of Pixel	Description
0–100	NDSI snow cover
200	Missing data
201	No decision
211	Night
237	Inland water
239	Ocean
250	Cloud
254	Detector saturated
255	Fill

**Table 2:** Performance criteria of streamflow simulation.

		Calibration						Validation					
		Daily				Monthly		Daily				Monthly	
		NSE	PBIAS	P-Fact	R-Fact	NSE	PBIAS	NSE	PBIAS	P-Fact	R-Fact	NSE	PBIAS
Tizi N’Isly	S1	0.51	−34.76	0.45	0.76	0.52	−34.61	0.33	−12.94	0.40	0.97	0.53	−12.74
	S2	0.52	−10.98	0.48	0.97	0.71	−10.87	0.49	−26.97	0.52	1.45	0.61	−26.79
Ait Ouchene	S1	0.59	−29.27	0.39	0.37	0.68	−29.07	0.55	−2.40	0.41	0.81	0.80	−2.22
	S2	0.62	−11.90	0.51	1.65	0.73	−11.78	0.63	−30.56	0.47	1.68	0.75	−30.31
Bine El Ouidane	S1	0.60	−9.43	0.52	0.72	0.84	−9.26	0.52	10.15	0.41	0.73	0.82	10.24
	S2	0.60	−17.56	0.55	1.15	0.69	−17.43	0.59	−7.73	0.49	1.18	0.79	−7.52
Tillouguite	S1	0.42	10.53	0.45	0.96	0.89	10.75	0.44	18.28	0.43	1.09	0.83	18.33
	S2	0.49	−6.94	0.52	1.38	0.89	−6.72	0.48	−3.79	0.55	1.89	0.84	−3.75
Overall performances	S1	0.53	−15.73			0.73	−15.55	0.46	3.27			0.74	3.40
	S2	0.56	−11.85			0.76	−11.70	0.55	−17.26			0.75	−17.09

**Table 3:** Optimal SWE-SCA thresholds providing the best estimates of SCA compared to MODIS.

Model	Ait Ouchene	Tillouguite
S1	5–15	10–25 mm
S2	10–20	10–30 mm

**Table 4:** Statistical criteria between simulated SWAT-SCA and MODIS-SCA.

	Timestep	NSE		PBIAS	
		S1	S2	S1	S2
Ait Ouchene sub-catchment	Daily	−0.4	0.14	−171	−21.11
	Weekly	−0.34	0.21	−170.25	−20.97
	Monthly	−0.57	0.23	−172.42	−22.31
Tillouguite sub-catchment	Daily	0.53	0.56	−14.38	−11.26
	Weekly	0.64	0.65	−14.12	−11.00
	Monthly	0.73	0.76	−14.26	−11.14
Oued El Abid basin-wide	Daily	0.49	0.54	−40.6	−31.48
	Weekly	0.62	0.68	−40.17	−31.05
	Monthly	0.69	0.75	−40.68	−31.56

**Figure A1:**
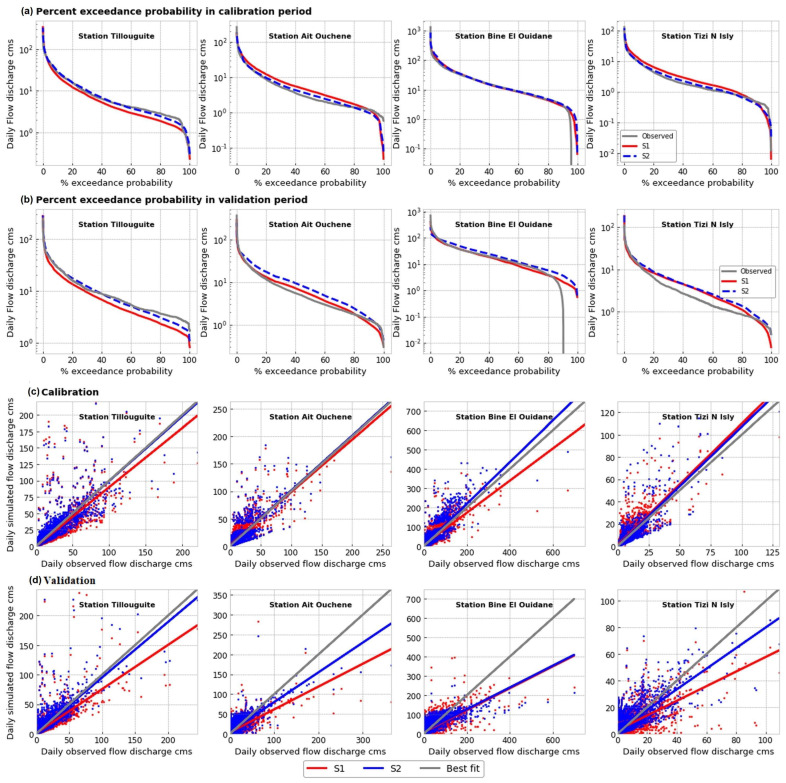
Percent exceedance probability for (**a**) calibration and (**b**) validation period and scatter plot between observations and simulations in (**c**) calibration and (**d**) validation.

**Figure A2:**
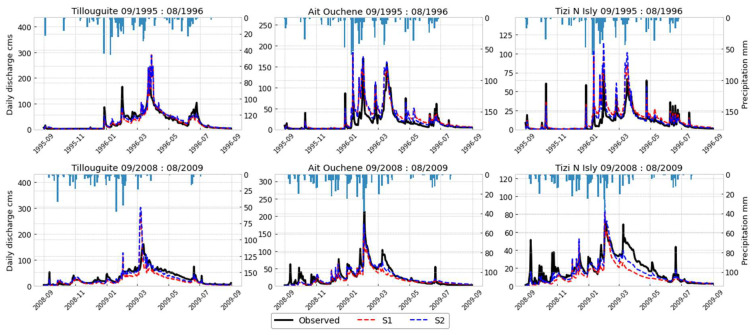
Hydrographs of simulated against observed daily streamflow for the years 1995–96 and 2008–09.

## Data Availability

Currently, meteorological and flow data are not openly accessible; inquiries should be sent to “https://www.marocmeteo.ma/ (accessed on 5 February 2022)”, and “http://www.abhoer.ma/ (accessed on 5 February 2022)”. MODIS snow cover products are freely provided by NASA National Snow and Ice Data Center “https://nsidc.org/ (accessed on 1 June 2022)”. The code that was modified and used to filter clouds from MODIS snow cover is available at “https://gitlab.inf.unibz.it/earth_observation_public/modis_snow_cloud_removal (accessed on 13 June 2022)”.

## Appendix A. SWAT Simulation of Streamflow

### Appendix A.1. Parameters Uncertainties

Table A1 shows the range of the 10% best-performing set of the two parameters from the last iteration. The values of TLAPS were set between −7.78 °C/km and −3.23 °C/km. The TLAPS results, however, confirm previous research in the area and demonstrate that temperature will decrease with elevation. For instance, Ref. [107] calibrated a SWAT model over the Nfis Basin in the High Atlas region. According to the findings, the TLAPS value was around −6.49 °C/km. Additionally, several studies in Morocco focused on the temperature lapse rate [85,93,106,108], which showed that the typical values fluctuate from year to year and from one season to another and are around −5.6 °C/km. Ref. [109] investigated eleven years of half-hourly climate measurements from the Joint International Laboratory LMI-TREMA (Télédétection et Ressources en Eau en Méditerranée semi-Aride) network of meteorological stations in the Marrakech High Atlas. According to their results, mean temperature lapse rates range from −3.67 °C/km to −5.21 °C/km monthly and, during the day, from −2.75 °C/km to −7.1 °C/km. Several studies conducted in different parts of the world showed similar outcomes [13,30,39,110]. Regarding the precipitation lapse, the obtained values for the parameter PLAPS ranged from 18.54 mm/km to 265.66 mm/km. It must be considered that the elevations of the Oued El Abid watershed range from 500 m in the lowlands to more than 3500 m in the mountain summits. Using this rate of lapse, the precipitation will be doubled. This is consistent with the findings of [48], who reports that while average annual precipitation in Morocco’s agricultural plains is only 500 mm, the Atlas Mountains, which rise above 3500 m in elevation, receive roughly twice that volume, mainly in the form of snow from November to mid-May. Ref. [107] found PLAPS around 444 mm/km in the basin of N’fis within the Marrakech High Atlas. Otherwise, the values found in other studies can vary from 5 mm/km to 623 mm/km [13,30,44,109,110]. Therefore, precipitation increases with elevation, but at varying rates. This indicates the non-linear relationships between precipitation and elevation, as confirmed by [49] in the Oum Er-Rbia catchment.

**Table A1:** Optimal values of lapse parameters.

Name of Parameter	Description	Changing Method	Range Min/Max	Fitted Value			
				Tizi Nisly	Tillouguite	Ait Ouchene	Bine EL Oudiane
PLAPS.sub	Precipitation lapse rate (°C/km)	Replace	−100/300	[146.14:200.94]	[265.66:169.06]	[84.59:54.37]	[29.55:18.54]
TLAPS.sub	Temperature lapse rate (mm/km)	Replace	−6/6	[−5.84:−3.71]	[−6.79:−3.88]	[−3.33:−7.78]	[−4.52:−2.90]

The next set of parameters that require calibration after lapse parameters are snow parameters, which describe the snow processes. For two configurations, lumped and spatially varying, the seven parameters under consideration are tuned in parallel against observed streamflow (see Figure 2). The top-10%-performing parameter sets for each configuration from the most recent iteration are displayed in Table A2. However, the best-performing ranges show substantial changes between S1 and S2. The analysis shows that uncertainties in the parameters vary largely among different models. There is a clear increase in parameter uncertainties in S2 compared to S1. In Tizi N’isly and Ait Ouchene, S2 tends to raise the snowfall temperature threshold, SFTMP. The increase in this parameter, which is responsible for the classification of precipitation into rain or snow based on air temperature, will allow for increased snow accumulation throughout the winter in this region. Additionally, S2 implies higher snowmelt factor SMTMP in Tizi N’isly and Ait Ouchene subbasins compared to the Tillouguite subbasins, where the model tends to increase the melt factor. By increasing the snowmelt factor, snow will not melt too early, which will result in less snow during the winter when the minimum air temperature is below zero. Snowmelt is expected to be more significant at the beginning and end of the season. However, it is expected that model S2 will simulate higher snow accumulation in the subbasins and higher snowmelt as a result of increased SFTMP and SMTMP.

**Table A2:** Calibrated SWAT snow parameters and their optimal values.

Scenario	Parameter	Description	Changing Method	Optimal Range			
				Tizi Nisly	Tillouguite	Ait Ouchene	Bine EL Oudiane
S1	TIMP.bsn	Snowpack temperature lag factor (-)	Replace	[0.43:0.24]			
	SFTMP.bsn	Snowfall temperature (°C)	Replace	[−0.32:−0.61]			
	SMTMP.bsn	Snowmelt base temperature (°C)	Replace	[0.05:−0.03]			
	SMFMX.bsn	Maximum melt rate for snow during year (mm H_2_O/°C-day)	Replace	[8.118:6.34]			
	SMFMN.bsn	Minimum melt rate for snow during the year (mm H_2_O/°C-day)	Replace	[1.34:0.13]			
	SNOCOVMX	Minimum snow water content that corresponds to 100% snow cover [mm]	Replace	[18.01:24.56]			
	SNO50COV	Fraction of snow volume represented by SNOCOVMX that corresponds to 50% snow cover	Replace	[0.1:0.9]			
S2	SUB_TIMP.sno	Snowpack temperature lag factor (-)	Replace	[0.62:0.14]	[0.46:0.15]	[0.58:0.22]	[0.44:0.04]
	SUB_SFTMP.sno	Snowfall temperature (°C)	Replace	[0.4: −0.34]	[−0.13:−0.76]	[0.15:−0.29]	[0.63:−0.65]
	SUB_SMTMP.sno	Snowmelt base temperature (°C)	Replace	[0.52:0.05]	[1.13:0.14]	[0.1:−0.2]	0.5:−0.89]
	SUB_SMFMX.sno	Maximum melt rate for snow during year (mm H_2_O/°C-day)	Replace	[8.23:6.48]	[6.91:5.21]	[8.32:4.51]	[6.92:4.12]
	SUB_SMFMN.sno	Minimum melt rate for snow during the year (mm H_2_O/°C-day)	Replace	[1.82:1.39]	[1.05:0.12]	[1.45:0.61]	[1.4:0.44]
	SUB_SNOCOVMX.sno	Minimum snow water content that corresponds to 100% snow cover [mm]	Replace	[12.67:18.40]	[15.58:31.30]	[16.92:24.65]	[12.11:28.49]
	SUB_SNO50COV.sno	Fraction of snow volume represented by SNOCOVMX that corresponds to 50% snow cover	Replace	[0.23:0.77]	[0.30:0.81]	[0.21:0.76]	[0.17:0.63]

The watershed parameters were finally calibrated in each configuration after multiple iterations. The range of the best-10%-performing parameter sets are presented in Table A3. The parameters concerning routing process, landscape, and groundwater are taken into consideration. Table A3 summarizes only parameters that are most sensitive to the model’s output. The ranges of both models are relatively similar since they overlap in most cases, which reveals the good significance of the parameters’ distribution. Nevertheless, the parameters’ uncertainties have been found larger in S2 compared to S1 in most of the parameters. Additionally, while certain model parameters may be reasonably improved using an optimization approach, others may not, making it difficult to determine whether the calibrated values are right. Future studies should focus on quantifying and reducing parameter uncertainty in semi-distributed models. Thus, the employment of a multi-objective formulation, where many variables are incorporated in the objective function, has demonstrated a good advantage for reducing uncertainty in parameters [111]. The use of various variables, both alone and in conjunction with streamflow measurements, has been demonstrated by several authors as a potential enhancer of the model’s performance, even permitting a further understanding of hydrological processes. However, because of the lack of ground observations and inadequate or low-quality data (i.e., missing, irregularly sampled, poorly validated, etc.), multi-objective calibration approaches represent a challenging issue in data-sparse locations. In many developing nations with no long-term planning for monitoring water resources or water management, the issue is increasingly obvious and dire. Therefore, a thorough comprehension of the current issues and creative management strategies are required. One of the most promising approaches is the use of recently developed earth observations [112]. Previous studies have demonstrated the efficacy of remotely sensed products to implement and calibrate hydrological models [113,114,115,116]. For example, several studies have used remotely sensed evapotranspiration [112,117], soil moisture [118], leaf area index [116,119], and snow water equivalent [44] to calibrate the SWAT model.

**Table A3:** Calibrated SWAT watershed parameters and their optimal values.

Parameter	Description	Changing Method	Model	Fitted Value			
				Tizi Nisly	Tillouguite	Ait Ouchene	Bine EL Oudiane
CANMX.hru	Maximum canopy storage (mm H_2_O)	Replace	S1	[12.07, 15.81]	[3.22, 5.53]	[10.18, 14.61]	[14.81, 20.06]
			S2	[8.45, 14.69]	[2.25, 5.14]	[7.11, 13.58]	[10.34, 18.65]
CH_N2.rte	Manning’s “n” value for the main channel (-)	Replace	S1	[0.05, 0.07]	[0.03, 0.04]	[0.06, 0.09]	[0.03, 0.04]
			S2	[0.01, 0.14]	[0.01, 0.08]	[0.02, 0.18]	[0.01, 0.08]
CH_K2.rte	Effective hydraulic conductivity in main channel alluvium (-)	Replace	S1	[12.77, 19.41]	[8.22, 10.99]	[10.33, 11.85]	[51.52, 75.68]
			S2	[9.21, 20.99]	[5.93, 11.88]	[7.45, 12.81]	[37.16, 81.81]
CN2.mgt	Curve number (-)	Relative	S1	[−0.15, −0.29]	[−0.22, −0.28]	[−0.19, −0.24]	[−0.15, −0.22]
			S2	[−0.12, −0.47]	[−0.18, −0.45]	[−0.16, −0.39]	[−0.11, −0.24]
OV_N.hru	Manning’s “n” value for overland flow (-)	Replace	S1	[0.07, 0.11]	[ 0.05, 0.14]	[0.01, 0.02 ]	[0.01, 0.04]
			S2	[0.09, 0.16]	[ 0.01, 0.17]	[0.01, 0.08 ]	[0.01, 0.03]
SLSOIL.hru	Slope length for lateral subsurface flow (m)	Replace	S1	[11.60, 16.32]	[20.61, 24.34]	[11.75, 12.86]	[33.12, 49.49]
			S2	[10.07, 18.90]	[17.89, 28.19]	[10.20, 14.89]	[28.75, 57.3]
SOL_AWC.sol	Available water capacity of the soil (mm H_2_O mm soil-1)	Relative	S1	[0.03, 0.09]	[0.29, 0.37]	[−0.09, −0.12]	[0.06, 0.08]
			S2	[0.02, 0.12]	[0.19, 0.49]	[−0.06, −0.16]	[0.04, 0.11]
ESCO.hru	Soil evaporation compensation factor (-)	Replace	S1	[0.64, 0.96]	[0.62, 0.81]	[0.60, 0.96]	[0.71, 0.98]
			S2	[0.67, 0.86]	[0.65, 0.73]	[0.63, 0.96]	[0.75, 0.94]
SOL_CRK.sol	Maximum crack volume of the soil profile expressed as a fraction of the total soil volume	Relative	S1	[0.02, 0.03]	[0.10, 0.16]	[0.11, 0.17]	[0.08, 0.12]
			S2	[0.02, 0.12]	[0.06, 0.14]	[0.07, 0.19]	[0.08, 0.15]
LAT_TTIME.hru	Lateral flow travel time (day)	Replace	S1	[2.60, 3.00]	[10.01, 11.11]	[8.62, 10.74]	[14.24, 18.98]
			S2	[2.77, 2.91]	[10.66, 10.78]	[9.18, 10.42]	[15.17, 18.41]
ALPHA_BF.gw	Baseflow alpha factor (-)	Replace	S1	[0.50, 0.61]	[0.29, 0.51]	[0.32, 0.36]	[0.29, 0.34]
			S2	[0.39, 0.65]	[0.23, 0.54]	[0.25, 0.38]	[0.23, 0.36]

### Appendix A.2. Graphical Comparison between Simulations and Streamflow

The % exceedance probability of the observed and simulated values for each station during the calibration and validation periods are shown in Figure A1a,b, respectively. This kind of plot, which many authors suggest as a tool for obtaining a useful overview for the evaluation of the model [76], shows how accurately the model reproduces the frequency of measured flows [120]. The % exceedance probability was plotted using log10 normal probability to highlight the visibility of low values. Figure A1c,d illustrates the scatter plot and linear regressions between observed and simulated data in both calibration and validation phases, respectively. Generally, the frequency of the daily simulated streamflow showed good agreement with the observations in both models S1 and S2 in the calibration period (Figure A1a,b), while, in the validation period, Figure A1b,d reveals an important increase in the discrepancies between the models and measurements of daily streamflow. However, for both the calibration and validation periods, we can see in Figure A1a,b that model S1 underestimated the low and median values of streamflow at the Tillouguite station, while the streamflow of Tizi Nisly and Ait Ouchene has been overestimated at median ranges by S1. The model S2 effectively improves the simulation of streamflow in the calibration phase for all stations as well as in the validation phase of Tillouguite. In contrast, the S2 model has slightly less accurate performances when considering the validation of Tizi N’isly and Ait Ouchene, particularly in median values.

Figure A2 illustrates the hydrographs of simulated and observed streamflow during the best-performing year in calibration (1995/96) and in validation (2008/09). As can be seen, SWAT models generally agreed well with discharge in most events of the year 1995/96; in contrast, models performed worse in the period of 2008/09, especially in the recession period. The models were able to properly capture some peaks but not others. In contrast, recession limb and base flow have been improved in model S2. However, the rise in the snowfall/snowmelt factor allowed more snow accumulation in winter; this water melts in spring and joins rivers through the root zone and groundwater. Therefore, S2 showed more consistency with observations, and this is more obvious in the Tillouguite basin as well as Tizi N’isly for the year 2008/09, where S2 tends to increase median flow and shows slight increased peaks compared to S1. SWAT generally demonstrates its ability to satisfactorily predict streamflow. The catchment of Oued El Abid is dominated geologically by the presence of large Jurassic and Lias aquifers. These aquifers, dominated by limestones, are giving rise to many sinkholes and springs. Therefore, the modelling of hydrological processes in this study was a very hard task, due to the difficulties in getting the best model to fit observations. As discussed previously, the Crack Flow module was modified to recall cracks independently of soil moisture conditions. Nevertheless, modifying the Crack Flow module resulted in a higher infiltration rate (not shown in this paper), which is also confirmed by [56]. The amount of infiltrated water will eventually increase quick subsurface flow and baseflow, while surface runoff and evapotranspiration decrease. In addition, observed large flows show rapid swings, which seems to indicate that runoff can play a very important role, even in karstic regions, probably because the infiltration coefficients are not sufficient to fully absorb high intensity rains, especially in the winter and spring seasons [50,121]. The uncertainties of the parameters related to deep aquifer recharge and baseflow calculations still require further investigations in a karst catchment. However, for a better understanding of the relationships between lateral, baseflow, and streamflow, a coupled modelling of surface water and groundwater in this area is recommended [122]. Furthermore, the implementation of such a system could be improved by using additional field data (spring flow records, sinkhole diameter or their exact locations, etc.). The figure below shows the evaluation of the snow simulation using MODIS-SCA.

### Appendix A.3. Impact of Different Configurations on Water Balance

The SWAT model simulation suggests that most of the runoff occurs as lateral flow and baseflow, and only very little amounts are contributed from the surface runoff (Figure A3). Generally, surface runoff in the catchment varies from a very small fraction close to zero, with the maximum value being recorded in the winter season. Moreover, spring appears to be the better season for baseflow and lateral formation. The spatiotemporal variation given by the model perfectly matches the assumption made by many researchers, which suggests more lateral and baseflow in the region [6,8,50,104,121]. However, Ref. [104] claims that the reason for this region’s decreased contribution from surface runoff is the quantity of snowpack there. Furthermore, the major area of the watershed is characterized by permeable and partially permeable terrains [50]. The precipitation takes advantage of this geological characteristic to infiltrate and feed the hydro-systems. However, changes in snow dynamics will cause changes in watershed parameters, which will in turn make some changes in SWAT water balance at the spatial and temporal units. Figure A3 represents the monthly variation of water balance components regarding both configurations. However, differences across projects are significant in most of the water balance components. The S2 project increases snowfall in the Ait Ouchene sub-catchment, permitting more water to be retained in the watershed as snow and soil moisture. As a result, the S2 project has simulated a greater quantity of snowfall/snowmelt, allowing for significant water partitioning between sub-catchments. Changes occur on both a spatial and temporal scale. However, actual evapotranspiration, one of the major fluxes, has increased. In addition, S2 boosted lateral flow and baseflow while decreasing surface runoff somewhat in the peak values. This is because more precipitation was classified as snow by raising the snowfall temperature threshold in this area. On the other hand, percolation flux has not changed much.
